# Detection of immunoreactive proteins of *Escherichia coli*, *Streptococcus uberis*, and *Streptococcus agalactiae* isolated from cows with diagnosed mastitis

**DOI:** 10.3389/fcimb.2023.987842

**Published:** 2023-02-10

**Authors:** Anna Dobrut, Dagmara Wójcik-Grzybek, Agata Młodzińska, Dorota Pietras-Ożga, Katarzyna Michalak, Aleksander Tabacki, Urszula Mroczkowska, Monika Brzychczy-Włoch

**Affiliations:** ^1^ Department of Molecular Medical Microbiology, Chair of Microbiology, Jagiellonian University Medical College, Krakow, Poland; ^2^ Department of Experimental Physiology, Chair of Physiology, Jagiellonian University Medical College, Krakow, Poland; ^3^ Bioidea Company, Warsaw, Poland; ^4^ Department of Epizootiology and Clinic of Infectious Diseases, University of Life Sciences, Lublin, Poland; ^5^ Omniwet Veterinary Clinic, Orzesze, Poland; ^6^ Egida Veterinary Clinic, Wizna, Poland

**Keywords:** mastitis, cattle, immunogenic proteins, mastitis diagnostics, bioinformatic analysis, *Escherichia coli*, *Streptococcus uberis*, *Streptococcus agalactiae*

## Abstract

**Introduction:**

Mastitis is a widespread mammary gland disease of dairy cows that causes severe economic losses to dairy farms. Mastitis can be caused by bacteria, fungi, and algae. The most common species isolated from infected milk are, among others, *Streptococcus* spp., and *Escherichia coli*. The aim of our study was protein detection based on both *in silico* and *in vitro* methods, which allowed the identification of immunoreactive proteins representative of the following species: *Streptococcus uberis*, *Streptococcus agalactiae*, and *Escherichia coli*.

**Methods:**

The study group included 22 milk samples and 13 serum samples obtained from cows with diagnosed mastitis, whereas the control group constituted 12 milk samples and 12 serum samples isolated from healthy animals. Detection of immunoreactive proteins was done by immunoblotting, while amino acid sequences from investigated proteins were determined by MALDI-TOF. Then, bioinformatic analyses were performed on detected species specific proteins in order to investigate their immunoreactivity.

**Results:**

As a result, we identified 13 proteins: 3 (molybdenum cofactor biosynthesis protein B, aldehyde reductase YahK, outer membrane protein A) for *E. coli*, 4 (elongation factor Tu, tRNA uridine 5-carboxymethylaminomethyl modification enzyme MnmG, GTPase Obg, glyceraldehyde-3-phosphate dehydrogenase) for *S. uberis*, and 6 (aspartate carbamoyltransferase, elongation factor Tu, 60 kDa chaperonin, elongation factor G, galactose-6-phosphate isomerase subunit LacA, adenosine deaminase) for *S. agalactiae*, which demonstrated immunoreactivity to antibodies present in serum from cows with diagnosed mastitis.

**Discussion:**

Due to the confirmed immunoreactivity, specificity and localization in the bacterial cell, these proteins can be considered considered potential targets in innovative rapid immunodiagnostic assays for bovine mastitis, however due to the limited number of examined samples, further examination is needed.

## 1 Introduction

Bovine mastitis is a major disease that affects dairy cattle worldwide, generating millions of dollars in losses for cattle breeders (Hogeveen et al., 2011). Mastitis results from inflammation of the mammary glands, and depending on the nature of the causative pathogen and on the age, breed, immunological health, and lactation state of the animal, it can take the forms of subclinical, clinical, or chronic infections. The most common clinical symptoms of mastitis are visible and palpable signs of swelling, pain, and redness of the udder, reduced milk production, altered milk appearance, and increased temperature or fever (Wilson et al., 1997; Kulkarni and Kaliwal, 2013). The chronic form is the least frequent form and leads to persistent inflammation of the mammary gland. According to the literature, the frequency of the clinical form of mastitis varies among different countries and ranges from 12 to 30% (Kalińska et al., 2018). On the other hand, the subclinical form involves the highest costs associated with difficulties in detection due to the lack of visible symptoms (Yalçin, 2000). Mastitis leads to a reduction in the volume of obtained milk and deterioration of its quality. It also generates costs related to the treatment of sick animals as well as costs associated with the need for isolation of sick animals for the duration of treatment. Furthermore, isolated cows produce a reduced amount of milk, which significantly reduces financial profits (Hogeveen et al., 2011). It is estimated that the monthly economic losses generated by clinical mastitis can reach up to 76,000 USD/farm/month (He et al., 2020).

Currently, the most common method for rapid detection of mastitis is based on somatic cell count (SCC) in quarter milk, and 250 × 10^3^ cells/ml is considered a critical value (Rushton et al., 2018). Undoubtedly, the most important advantage of this “gold standard” method is the possibility of rapid detection of mastitis in animals examined directly in the field. It leads to immediate isolation of the infected cow from the herd and allows the introduction of empirical antibiotic therapy. However, a significant limitation of the SCC test is the inability to identify the etiological factor that causes the infection (Naif Alhussien and Kumar Dang, 2018). Empirical therapy, which is introduced when the etiological agent responsible for the disease is not identified, can contribute to increased resistance to antibiotics. Therefore, the determination of the pathogen present in infected milk is crucial for the selection of targeted antibiotic therapy, which can lead to a decrease in this worldwide adverse phenomenon (Oliver and Murinda, 2012). Therefore, supported by the biotechnology industry, scientists have undertaken numerous studies, which are aimed at the development of innovative tests that allow the precise identification of bacterial species (Viguier et al., 2009).

Among infectious pathogenic species, the most frequent are *Staphylococcus aureus*, *Corynebacterium bovis* and *Mycoplasma bovis*, while the predominant environmental pathogens are *Streptococcus uberis*, *Streptococcus dysgalactiae*, *Enterococcus* spp., *Escherichia coli*, *Klebsiella pneumoniae*, and *Enterobacter* spp. Interestingly, in recent years, algae and fungi have been increasingly isolated from mastitic milk (Lassa et al., 2013; Dufour et al., 2019).

The most common method for the detection of the etiological agent causing mastitis in cows is cultivation in the appropriate cultivation medium. This inexpensive method not only allows identification of pathogens, but also allows the multiplication of bacteria for further, more specific, characterization, including the determination of the resistance profile, and does not require expensive or specialized equipment. On the other hand, the limitations include, being time-consuming, low sensitivity and specificity, frequent difficulty in unambiguous interpretation, and most of all, the inability to perform the analysis in non-laboratory conditions, which is a significant limitation factor for field veterinarians (Hogan et al., 1999; Koskinen et al., 2010).

In recent decades, molecular methods have gradually gained in popularity in bacteriological diagnostics, with particular emphasis on polymerase chain reaction (PCR) (Koskinen et al., 2010). In parallel, serological methods based on the antigen-antibody reaction, such as enzyme-linked immunosorbent assay (ELISA), have been developed. Their common advantages are high selectivity and specificity, as the waiting time for the results is shorter in comparison to classical cultivation. Nevertheless, its crucial limitation is the inability to perform the tests outside of the laboratory equipped with specialized equipment (Kano et al., 2016). A promising alternative to these methods, which does not demonstrate the limitation mentioned above, is the immunochromatographic assay (lateral flow assay, LFA). This rapid test allows the detection of pathogens in biological samples (milk, blood, stool) within a few minutes, and moreover, it does not require specialized equipment, it is easy to perform, and it is simple to interpret. But what is most important is that the lateral flow assay can be performed directly in the field – not only by the veterinarian, but also by the cattle breeders. Furthermore, the low costs, compared to other molecular and serological methods, make this technique more accessible (Koczula and Gallotta, 2016).

In this paper, our objective was to detect the immunoreactive protein particular for three species causing mastitis in cattle: *E. coli*, *S. uberis*, and *S. agalactiae*, which demonstrates the basis of which they can be considered as potential biomarkers in an innovative immunochromatographic assay for the rapid diagnosis of cattle mastitis.

## 2 Materials and methods

### 2.1 Sampling

The study group included 22 bovine milk samples (50 ml of each sample) from infected quarters, which were isolated from cattle with clinical symptoms of mastitis such as swelling, heat, hardness, redness or pain of the udder, watery appearance, flakes, clots or pus in milk, increased body temperature, and/or lack of appetite, in the course of the procedure of routine submission of milk from different regions of Poland: Omniwet Veterinary Clinic, Orzesze (south west) and Egida Veterinary Clinic, Wizna (north east). Cows included in the investigation came from different cowsheds – both stationary and free-standing cowsheds. Additionally, 13 blood samples for serum isolation were collected through a single jugular venipuncture during routine diagnostic tests to identify mastitis. Serum isolation was carried out according to Tuck et al. (Tuck et al., 2009). Blood samples were collected from the same cows from which the milk was collected. The aim of milk collection from infected cows, apart from obtaining bacterial strains for protein isolation, was identification of the etiological agent causing mastitis. This identification is the basis of which serum samples collected from the individual cow could be classified as EC-positive (serum sample obtained from a cow with diagnosed mastitis caused by *E. coli*), SU-positive (serum sample obtained from a cow with diagnosed mastitis caused by *S. uberis*), GBS-positive (serum sample obtained from a cow with diagnosed mastitis caused by *S. agalactiae*) and then used for detection of immunoreactive proteins representative for *E. coli*, *S. uberis*, and *S. agalactiae*. The sera studied had been pooled for either *E. coli* and *S. uberis*, whereas there was only one GBS-positive serum sample for *S. agalactiae*.

The control group constituted 12 milk samples and 12 serum samples obtained from 12 cows (**Table 1**), which were collected by veterinarians during routine examinations to determine typical biochemical parameters in healthy animals. The health status of the animals was determined on the basis of physical parameters, such as the absence of edema or redness of the udder, as well as biochemical parameters by determining the number of somatic cells (the average number of somatic cells among the animals included in the study was 160.000/ml). The studied herds were assessed by the Polish Federation of Cattle Breeders and Milk Producers, which requires monthly reporting of milk parameters, such as the number of somatic cells per milked animal. The purpose of milk collection from healthy animals was exclusion of ongoing infection, and on its basis, a classification of serum samples to control group was done. The aim of inclusion of serum samples from healthy animals was examination of protein specificity. Additionally, to exclude cross-reactivity among *Streptococcus* isolates, a milk sample (n=1) from a cow infected with *S. dysgalactiae* was subjected to investigation. Milk and serum samples were stored at -80˚C until further analyses. According to the opinion of 1st Local Ethics Committee for animal experimentation the consent for the research obtained for the project was not required.

**Table 1:** List of milk and serum samples included in the detection of immunoreactive proteins representative for *Escherichia coli*, *Streptococcus uberis* and *Streptococcus agalactiae*.

Species	Milk (cow no.)	Serum (cow no.)
*Streptococcus uberis*	5, 6, 7, 8, 9, 10, 11, 12, 13, 14, 15	7, 9, 10, 11, 12, 14
Total number	n=11	n=6
*Escherichia coli*	1, 2, 3, 4	2, 3
Total number	n=4	n=2
*Streptococcus agalactiae*	16	16
Total number	n=1	n=1
Other species	17, 18, 19, 20, 21, 22	19, 20, 21, 22
Total number	n=6	n=4
Healthy animals (negative control)	23, 24, 25, 26, 27, 28, 29, 30, 31, 32, 33, 34	23, 24, 25, 26, 27, 28, 29, 30, 31, 32, 33, 34
Total number	n=12	n=12

### 2.2 Bacterial culture and identification

100 µl of each milk sample, both positive and negative, was propagated on Columbia sheep blood agar (Difco) under aerobic conditions at 37°C for 24 h. Furthermore, bacterial isolates that were preliminarily classified as *E. coli* were grown on MacConkey agar (Oxoid) under aerobic conditions at 37°C for 24 h. Next, colonies with morphology corresponding to the species sought were isolated from the medium and subjected to further, more specific, analyses, including cultivation on Granada Agar (bioMérieux) dedicated to the detection of β-hemolytic *S. agalactiae*, CHROMagar™ Mastitis GP (CHROMagar™) for the diversification of the most common Gram-positive species in mastitis, Christie–Atkins–Munch-Peterson test (CAMP test), catalase test, analytical profile index (API test) and matrix assisted laser desorption and ionisation time-of-flight (MALDI-ToF) identification (Nonnemann et al., 2019).

### 2.3 Protein isolation and detection

The isolation of bacterial proteins was carried out according to the procedure of Park et al. (Park et al., 2020), whi.ch is the protocol described by Fang and Oliver (Fang and Oliver, 1999) with modifications, and included culture of bacterial isolates in Tryptic Soy Broth (TSB; Becton-Dickinson) at 37°C for 24 h. The bacterial culture was then centrifuged (30 min, 731 x g). Subsequently, the supernatant was removed and bacterial pellets were suspended in 3 ml of phosphate buffered saline (PBS; Thermo Fisher) and centrifuged for 20 minutes (731 x g). After repeating these steps three times, the wet mass of the bacteria was weighed and, for each 30 mg of the wet mass of cells, 100 µl of 0.2% sodium dodecyl sulphate (SDS; Sigma Aldrich) was added. The samples were incubated at 43°C for 1 h. The material was then centrifuged (20 min, 731 x g) and the supernatant of protein samples was collected. The remaining pellet was cultured in Columbia sheep blood agar medium (Difco) at 37°C for 18 h to confirm that the protein isolation procedure did not disrupt the bacterial cell wall. Therefore, bacteria grown on medium suggested that only cell surface-associated proteins were isolated. The concentration of the proteins was estimated using the Pierce™ BCA Protein Assay Kit (Thermo Fisher Scientific). The prepared protein samples were aliquoted and stored at −20 °C until use.

For protein separation, 10 µl of each sample was mixed with Laemmli buffer and applied to 4–20% gels (BIO-RAD) according to Laemmli (Laemmli, 1970). After sodium dodecyl sulfate–polyacrylamide gel electrophoresis (SDS-PAGE), which was performed at 80 V for 20 minutes, and then at 120 V for 40 minutes, the gels were washed three times in deionized water (diH_2_O) and stained with Coomassie Brilliant Blue (BIO-RAD) or directly transferred to a polyvinylidene difluoride membrane (Millipore) for immunoblotting. Protein transfer was carried out at 135 V for 90 minutes in the Mini-PROTEAN Tetra Cell apparatus (BIO-RAD). Afterward, the membranes were blocked in 1% bovine serum albumin (BSA; Thermo Fisher) in phosphate buffered saline with 0.05% Tween-20 (Thermo Fisher) at room temperature for 1 hour on an orbital shaker (50 RPM). Membranes were rinsed three times washing for 5 minutes in PBS-T, the membranes were then incubated with the serum samples diluted in PBS-T (1:1000) at 37°C for 2 h and washed three times for 5 minutes in PBS-T. Next, membranes were incubated with sheep anti-bovine IgG secondary antibodies conjugated with horseradish peroxidase (HRP; Novusbio) in a 1:10 000 dilution in PBS-T at room temperature for 1 h. After washing three times for 5 minutes in PBS-T, chemiluminescence was developed using Pierce™ ECL Western Blotting Substrate (Thermo Fisher Scientific) and measured using the C-DiGit^®^ Blot Scanner (LI-COR, NE, USA). Subsequently, bands of interest (3 for *E. coli*, 4 for *S. uberis*, 6 for *S. agalactiae*), defined as common among individual species and not present (or barely reactive) among other species, were cut out from the gel and subjected to further analyses including protein identification

### 2.4 Protein identification

In order to identify proteins, which were cut from the electrophoretic gel, gel fragments were destained by methanol, reduced, and alkylated using dithiothreitol and iodoacetamide solutions. The gel fragments were then digested overnight with trypsin in 50 mM bicarbonate buffer at 37°C (Promega, Trypsin Gold, Mass Spectrometry Grade, Technical Bulletin). The resulting peptides were then extracted from the gel with water/acetonitrile/trifluoroacetic acid by triple elution (v:v 45:50:5). The peptide mixtures were purified and concentrated using C18 Zip-TIP pipette tips according to the manufacturer’s guidelines (Merck Chemicals, Billerica, MA, USA, PR 02358, Technical Note).To identify proteins, the purified peptide mixtures and the standard peptide solution were spotted on an Anchor Chip MALDI plate (Bruker, Bremen, Germany) and covered by 1 μL of α-cyano-4-hydroxycinnamic acid matrix (HCCA, Bruker, Bremen, Germany). Mass spectra were recorded in positive reflector mode within the 700–4000 m/z range using an Ultraflextreme MALDI TOF/TOF spectrometer (Bruker, Bremen, Germany) and the flexControl 3.3 software (Bruker, Bremen, Germany). The obtained spectra were smoothed and the baseline was corrected. The peak list generated in the flexAnalysis 3.0 software (Bruker, Bremen, Germany) for the signal-to-noise ratio greater than 3 was transferred to BioTools 3.2 (Bruker, Bremen, Germany), and compared to Mascot 2.2 software (Matrix Science, Boston, MA, USA) using the Swiss-Prot database (www.uniprot.org) restricted to the taxonomy ‘bacteria’ with a maximum error of 0.3 Da, carbamidomethylation of cysteine as an obligatory modification, and trypsin as a cutting enzyme. The spectra were compared due to their similarity to the peptide mass fingerprint, which is characteristic for individual proteins. In this case, the number of overlapping peptide signals formed after digestion was used to compare the data with those present in the annotated protein sequence database. The results with a Mascot score greater than 61 were considered statistically significant (p ≤ 0.05); otherwise, the fragment ion spectra of the chosen peptides were recorded in LIFT mode and combined with the objective of MALDI TOF/TOF identification. The protein score is -10*Log(P), where P is the probability that the observed match is a random event. The higher the score value, the better the identification made.

### 2.5 Bioinformatic analysis

In order to investigate the structure, function and immunogenicity of the predicted proteins, multiple bioinformatic analyses were performed, including prediction of subcellular localization, signal peptides, antigenicity, and classical and non-classical proteins. In addition, selected proteins were subjected to linear B-cell epitope identification, protein data bank (PDB) model search and identification of conformational B-cell epitopes. The subcellular localizations of the proteins were predicted with PsortB 3.0 (Yu et al., 2010) and the Bologna Unified Subcellular Component Annotator (BUSCA) server (http://busca.biocomp.unibo.it/), separately for Gram-positive and Gram-negative bacterial proteins. The subcellular localizations were labeled with gene ontology (GO) annotation, extracellular as GO: 0005615 and cytoplasm GO: 0005737. Prediction of protein functions included prediction of signal proteins [PRED-LIPO (Bagos et al., 2008)], classical secretory proteins [SignalP (Almagro Armenteros et al., 2019)] or non-classical secretory proteins: for Gram-positive species [Secretome (Bendtsen et al., 2004)] and Gram-negative species [PncsHub (Dai et al., 2022)]. The classical and non-classical proteins were predicted with a default setting score of 0.45 and 0.5, respectively. Classical secretory proteins are proteins that are secreted outside the cell by signal peptides. Non-classical secretory proteins are proteins without signal peptides with different pathways of secretion outside the cell. To identify the most highly antigenic proteins, the *in silico* prediction was performed with the use of the VaxiJen (Doytchinova and Flower, 2007) software. Antigenic prediction was adjusted with a default setting score of 0.4, and those proteins with a score ≥0.4 were considered antigenic proteins. B-cell epitopes of antigenic proteins were identified with the use of Antibody Epitope Prediction in the Immune Epitope Database (IEDB) (Vita et al., 2015). Conformational B-cell epitopes were identified with the following steps: for each protein sequence, the PDB template was identified with the use of the SWISS-MODEL Workspace (Kiefer et al., 2009) including PDB IDs, next, the best template (with the higher identity) was chosen to build the model. The selected PDB IDs were then used for prediction of linear and discontinuous conformational B-cell epitopes with ElliPro software (Ponomarenko et al., 2008).

## 3 Results

### 3.1 Bacterial identification

**Figure 1:**
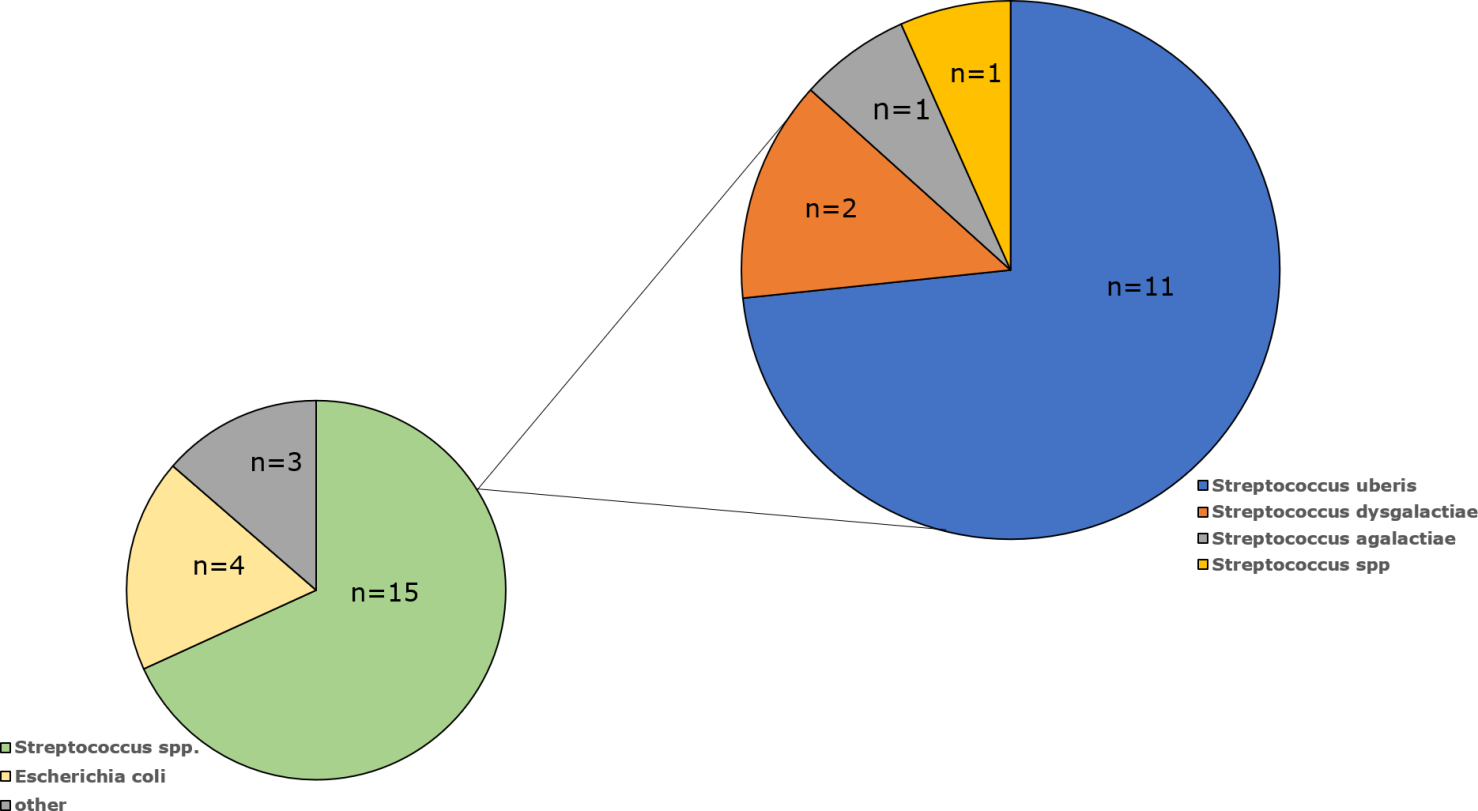
Percentage of bacterial isolates isolated from milk of cows diagnosed with mastitis by species.

Among the milk samples isolated from cows with mastitis, the species or genus has been confirmed for 22 isolates (**Table 1**). The most common were *Streptococcus* spp. (n=15; 68.3%), followed by *E. coli* (n=4; 18.2%), *Enterococcus cecorum* (n=1; 4.5%), *Aerococcus viridans* (n=1; 4.5%) and *Enterobacter cloacae* (n=1; 4.5%). Among the *Streptococcus* genus, *S. uberis* dominated as it was present in 11 samples (73%), *S. dysgalactiae* was detected in two specimens (13%), while *S. agalactiae* was present in only one examined milk sample (7%). One *Streptococcus* isolate (7%) was not identified at the species level (**Figure 1**).

### 3.2 Protein detection

**Figure 2:**
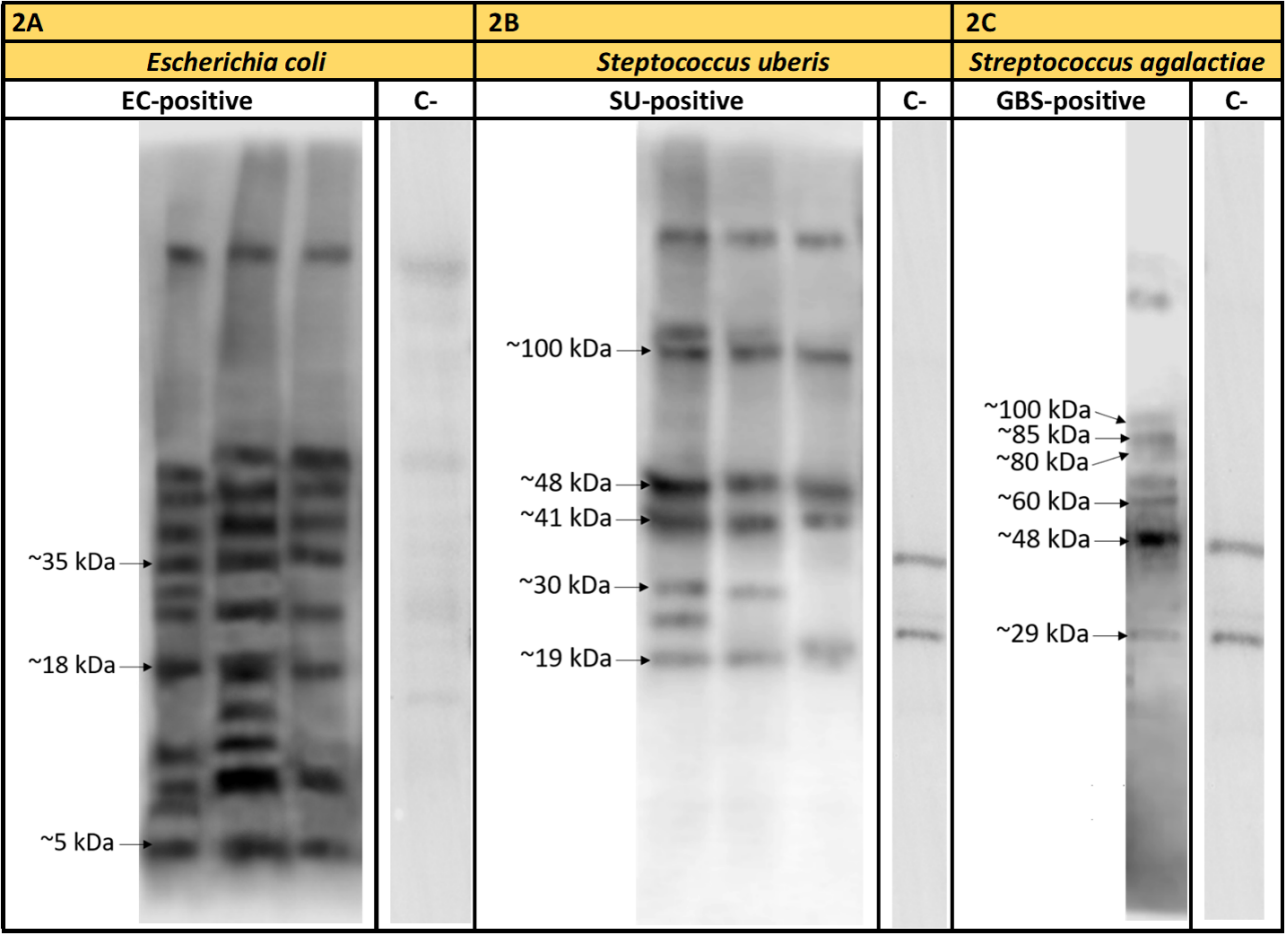
Exemplary results of immunodetection of proteins representative of *Escherichia coli* (strains no: 1, 2, 3) tested in the presence of sera from cows with diagnosed mastitis caused by *Escherichia coli* (EC-positive – sera no: 2, 3) **(2A)**; *Streptococcus uberis* (strains no: 7, 8, 9) tested in the presence of sera from cows with diagnosed mastitis caused by S. uberis (SU-positive –sera no: 7, 9, 10) **(2B)** and *Streptococcus agalactiae* (strain no: 16) tested in the presence of sera from cows with diagnosed mastitis caused by S. agalactiae (GBS-positive – serum no: 16) **(2C)**. Legend: C- – serum samples (sera no: 23, 29, 31) obtained from healthy animals.

**Figure 3:**
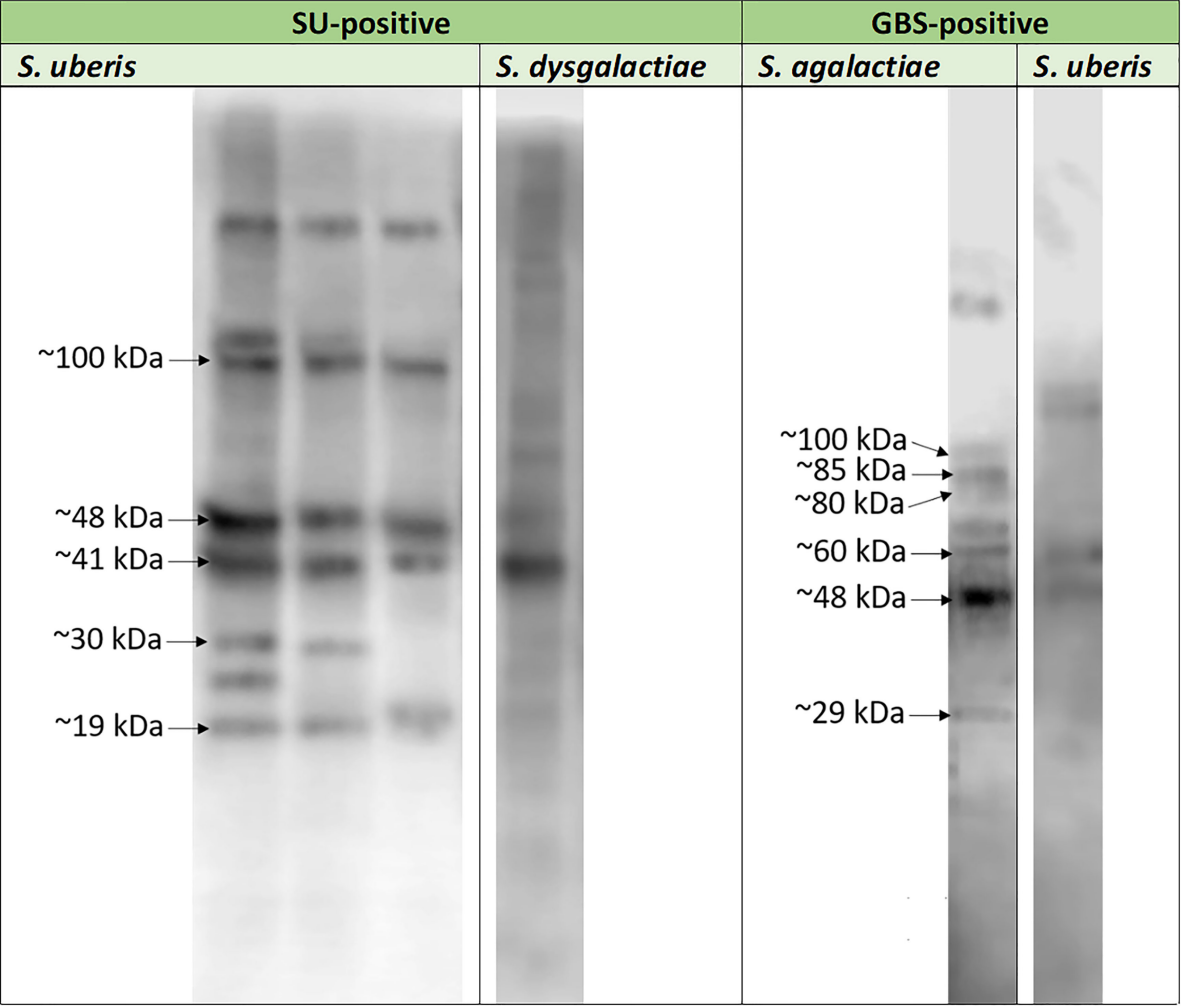
Exemplary results of the cross-reactivity detection among *Streptococcus agalactiae* (strain no: 16), *Streptococcus uberis* (strains no: 7, 8, 9) and *Streptococcus dysgalactiae* (strain no: 17) strains investigated in the presence of serum sample obtained from cow with diagnosed mastitis caused by *Streptococcus agalactiae* (GBS-positive; serum no: 16) and pooled serum samples isolated from animals with diagnosed mastitis caused by *Streptococcus uberis* (SU-positive; sera no: 7, 9, 10) bovine serum samples.

Protein detection was carried out for three representative *E. coli* isolates, three *S. uberis* isolates, and one *S. agalactiae* isolates. Particular isolates were chosen randomly. Out of approx. 16 *E. coli* proteins immunoreactive with pooled EC-positive serum samples (no: 2, 3), three, with molecular masses of approx. 5 kDa (EC1), 18 kDa (EC2), and 35 kDa (EC3), were chosen for further analyses (**Figure 2A**). However, out of approx. 14 *S. uberis* proteins, which reacted with pooled SU-positive serum (no: 7, 9, 10), five bands were subjected to sequencing. Their molecular masses were as follows: approx. 19 kDa (SU4), 30 kDa (SU3), 41 kDa (SU5), 48 kDa (SU1), and 100 kDa (SU2) (**Figure 2B**). The lowest immunoreactivity of proteins was detected for *S. agalactiae* (group B streptococcus, GBS). Six proteins immunoreactive with GBS-positive serum (no: 16) with weights of: approx. 29 kDa (GBS1), 48 kDa (GBS2), 60 kDa (GBS3), 80 kDa (GBS8), 85 kDa (GBS4), and 100 kDa (GBS7) were subjected to further investigation (**Figure 2C**). Furthermore, the proteins mentioned above did not react or barely reacted with negative samples. We also did not observe cross-reactivity of most protein bands among the bacterial isolates obtained from the animals with confirmed mastitis caused by *Streptococcus* (*S. uberis*, *S. agalactiae*, *S. dysgalactiae*) (**Figure 3**).

### 3.3 Protein identification

The mass spectra obtained for selected electrophoretic strips were collected, base line corrected, and compared with those of the Swiss-Prot database. During the protein identification, we obtained positive results that led to the assignment of 13 proteins with a significance threshold p ≤ 0.05, of which 6 were isolated from *S. agalactiae*: aspartate carbamoyltransferase (GBS 1), elongation factor Tu (GBS 2), 60 kDa chaperonin (GBS 3), elongation factor G (GBS 4), galactose-6-phosphate isomerase subunit LacA (GBS 7), and adenosine deaminase (GBS 8), 4 originated from *S. uberis*: elongation factor Tu (SU 1), tRNA uridine 5-carboxymethylaminomethyl modification enzyme MnmG (SU 3), GTPase Obg (SU 4), glyceraldehyde-3-phosphate dehydrogenase (SU 5), and 3 came from *E. coli*: molybdenum cofactor biosynthesis protein B (EC 1), aldehyde reductase YahK (EC 2), and outer membrane protein A (EC 3). Differences between the molecular weight of proteins and the weight resulting from electrophoretic gels may be caused by the presence of multiple protein forms, their modification, or partial protein breakdown.

### 3.4 Bioinformatic analysis

Thirteen proteins were subjected to bioinformatic analysis to determine their immunogenicity and functionality. In the first stage of the analysis, the proteins were classified in terms of their location within the cell. As a result, 12 proteins were found in the cytoplasm (GO: 000573) and 1 protein (EC 3) was located in the outer membrane (GO: 0019867) of the subcellular compartments. In the next step, classical and non-classical proteins were identified. As a result, only in the EC 3 protein structure (outer membrane), a Sec signal peptide was identified and its presence determined the classification of this protein as classical. In addition, 9/13 proteins have been classified as non-classical proteins (known as secretome proteins), including again the outer membrane protein EC 3 and several cytoplasmic proteins (GBS 1, GBS 2, GBS 3, GBS 4, SU 1, and SU 5). Moreover, proteins were classified according to their antigenicity, resulting in 12/13 proteins being labeled as antigens (**Table 2**). The structures of 13 proteins were analyzed to identify B-cell epitopes. As a result, epitopes were found in all proteins, ranging in number from 1 to 22. The presence of B epitopes allowed for further identification of conformational epitopes in all proteins. First, the most identical PDB (template) protein structure was identified and a protein-template model was built. The template identity was in the range of 32-100% with Global Model Quality Estimation in range 0.39-0.96. The constructed models were then used to identify linear and conformational epitopes. As a result of the analysis, 2-10 linear epitopes and 3-8 conformational epitopes were obtained (**Table 3**).

**Table 2:** Bioinformatic prediction of proteins’ cell localization (Busca database), detection of signal peptides (PRED-LIPO), classical (SignaIP) and non-classical (Secretome, PncsHub) proteins, and prediction of antigenicity (VaxiJen).

ID	Protein	UniProt ID	MW [Da]	BUSCA^1^	PRED-LIPO	SignalP	Secretome*/PncsHub**	VaxiJen^2^
				Protein localization in cell	Prediction of lipoprotein signal peptides	Signal peptide predictions	Prediction of secreted proteins	Prediction of protective antigens
GBS_1	**Aspartate carbamoyltransferase**	Q3K148	34871	Cymiddlelasm	Other	N	Y	Antigen
GBS_2	**Elongation factor Tu**	Q3K1U4	43954	Cymiddlelasm	Other	N	Y	Antigen
GBS_3	**60 kDa chaperonin**	Q1JEL5	57245	Cymiddlelasm	Other	N	Y	Antigen
GBS_4	**Elongation factor G**	Q490W7	76538	Cymiddlelasm	Other	N	Y	Antigen
GBS_7	**Galactose-6-phosphate isomerase subunit LacA**	C0M8Q6	15123	Cymiddlelasm	Other	N	Y	Antigen
GBS_8	**Adenosine deaminase**	Q3K2D4	38067	Cymiddlelasm	Other	N	N	Non-antigen
SU_1	**Elongation factor Tu**	B9DRL9	43886	Cymiddlelasm	Other	N	Y	Antigen
SU_3	**tRNA uridine 5-carboxymethylaminomethyl modification enzyme MnmG**	Q5LXK0	70616	Cymiddlelasm	Other	N	Y	Antigen
SU_4	**GTPase Obg**	A2RJQ6	48205	Cymiddlelasm	Other	N	N	Antigen
SU_5	**Glyceraldehyde-3-phosphate dehydrogenase**	Q59906	35962	Cymiddlelasm	Other	N	Y	Antigen
EC_1	**Molybdenum cofactor biosynthesis protein B**	P0AF00	18768	Cymiddlelasm	Other	N	N	Antigen
EC_2	**Aldehyde reductase YahK**	P75691	38524	Cymiddlelasm	Other	N	N	Antigen
EC_3	**Outer membrane protein A**	P0A911	37292	Outer membrane	Sec signal peptide predicted. Most likely cleavage site: 1 - 21 [AQA-AP]	Y	Y	Antigen

MW, molecular weight values correspond to the MASCOT Search Result; Y, yes; N, no; Other, does not possess signal sequence; Sec signal peptide, possesses an amino-terminal extension of the secretory protein; ^1^BUSCA values ranged from 0.7 to 0.75; ^2^VaxiJen antigen prediction score ranged from 0.35 to 0.68.

**Table 3:** Bioinformatic prediction of proteins, identification of linear B cell epitopes (IEDB tools), detection of 3D model in PDB database (SWISS-MODEL), and identification of conformational B cell epitopes (ElliPro).

ID	Protein	UniProt ID	No. of B-cell epitopes	3D Structural Modelling				
				PDB templates	Identity %	GMQE	No. of conformational B cell epitopes (linear)	No. of conformational B cell epitopes (discontinuous)
			Prediction of B cell epitopes					
GBS_1	**Aspartate carbamoyltransferase**	Q3K148	10	6pnz.1.A	53.8%	0.81	1	5
GBS_2	**Elongation factor Tu**	Q3K1U4	11	6gfu.1.A	75.8%	0.81	6	5
GBS_3	**60 kDa chaperonin**	Q1JEL5	22	4v4o.1.B	65.1%	0.79	12	6
GBS_4	**Elongation factor G**	Q490W7	20	2xex.1.A	76.2%	0.84	4	8
GBS_7	**Galactose-6-phosphate isomerase subunit LacA**	C0M8Q6	1	4lfk.1.A	44.3%	0.82	0	3
GBS_8	**Adenosine deaminase**	Q3K2D4	11	3iar.1.A	32.3%	0.64	3	5
SU_1	**Elongation factor Tu**	B9DRL9	12	6i8r.1.A	76.8%	0.82	6	7
SU_3	**tRNA uridine 5-carboxymethylaminomethyl modification enzyme MnmG**	Q5LXK0	21	2zxi.1.A	52.2%	0.72	10	5
SU_4	**GTPase Obg**	A2RJQ6	15	1lnz.1.A	66.0%	0.67	8	5
SU_5	**Glyceraldehyde-3-phosphate dehydrogenase**	Q59906	11	6fzh.1.A	95.2%	0.94	4	4
EC_1	**Molybdenum cofactor biosynthesis protein B**	P0AF00	7	1r2k.1.A	100%	0.96	3	3
EC_2	**Aldehyde reductase YahK**	P75691	13	1uuf.1.A	99.7%	0.95	5	6
EC_3	**Outer membrane protein A**	P0A911	12	3nb3.1.A	99.4%	0.39	3	5

GMQE, Global Model Quality Estimation, calculated after building the model on selected template.

## 4 Discussion

Bovine mastitis is a worldwide economic issue that has been studied in many aspects for years. The aim of this paper was the detection of potential markers, which can find application in rapid multiplex immunochromatographic assay. Promising candidates for targets detected by monoclonal antibodies should demonstrate the following features: they should be conservative among species – to avoid false negative results – and species-specific – to avoid false positive results. This investigation included three bacterial species – *E. coli*, *S. uberis*, and *S. agalactiae* (Lassa et al., 2013; Dufour et al., 2019).

In most of the clinical cases of mastitis in our study, were caused by *Streptococcus* spp. The obtained results corresponded to the investigation described by Al-harbi et al. (Al-harbi et al., 2021), as well as to the North American cattle population (Schukken et al., 2009; Levison et al., 2016). Among the genus *Streptococcus*, the most frequently isolated species was *S. uberis*, which constitutes 44% of all samples studied. *S. uberis* is classified as an environmental mastitis pathogen, however, it can also take a contagious form, which can potentially originate from milk machine contamination (Zadoks et al., 2003). The results obtained in this paper corresponded to Australian studies, in which *S. uberis* was the most commonly cultivated bacterial species collected from milk samples (Dyson et al., 2022). Al-harbi et al. also described *S. uberis* as the most common species of *Streptococcus* spp., however, the percentage was fourfold lower compared to our results (Al-harbi et al., 2021). In the European cattle population, *S. uberis* is also described as the most common agent causing mastitis (Tenhagen et al., 2006; Botrel et al., 2010). It is no different in Poland (Malinowski et al., 2006).

In this study, the second most frequent bacterial species was *E. coli*, which belongs to the *Enterobacteriaceae* family, and is also defined as one of the most common causes of clinical mastitis in dairy cows (Schabauer et al., 2018). Infections caused by this species often occur in early lactation, and the reason for this phenomenon is the immunosuppression caused by metabolic deficiencies, which is more common at that time than in later lactation (Suriyasathaporn et al., 2000; Burvenich et al., 2003). Infection caused by *E. coli* through activation of IκB/NF-κB signaling quickly elicits strong inflammatory reaction. This leads to predominantly acute infection which is easy to detect (Günther et al., 2017; Goulart and Mellata, 2022). *E. coli* infections are commonly related to the summer season, when the temperatures and humidity are high. Moreover, during the summer season, as a result of heat stress, animals’ immunity decreases, which leads to an increase in infections. Beyond the summer season mastitis caused by *E. coli* is observed in herds with poor hygiene conditions, which is common for environmental pathogens (Rakib et al., 2020). In the studied population, the prevalence of *E. coli* was found in 16% of the samples. The results obtained corresponded to the Nepalese cow population, in which 16.5% of the animals were diagnosed with mastitis caused by *E. coli* (Bhandari et al., 2021). Abed et al. showed the same prevalence, detecting *E. coli* in 16.4% of the samples (Abed et al., 2021). Botrel et al. demonstrated the presence of *E. coli* in 16% of the French cattle population (Botrel et al., 2010).

Another species detected among the investigated milk samples was *S. agalactiae*, which is responsible for cases of contagious mastitis (Al-harbi et al., 2021). In our studies, *S. agalactiae* was present in one milk sample and constituted 4.5% of the examined bacterial isolate collection. The prevalence of *S. agalactiae* has decreased in recent decades. However, in the 1980s, it was the main cause of mastitis, reaching almost 50% (Keefe, 1997, Keefe, 2012; Al-harbi et al., 2021). The reason for the notable decrease in incidence is an effect of the introduction of the mastitis control program (Myllys et al., 1998; Pitkälä et al., 2004). Also, in Poland the decrease in the incidence of *S. agalactiae* has been observed in cattle (Malinowski et al., 2006). However, Sztachańska et al. showed that subclinical cases of *S. agalactiae* isolated in infected quarters reached 15.6%, but the high percentage was explained by the low efficacy of dry cow therapy in some herds (Sztachańska et al., 2016). Malinowski et al. reported that mastitis caused by *S. agalactiae* occurs in 2-25% of cows per year (Malinowski and Gajewski, 2009). Even though this contagious pathogen is not the main cause of bovine mastitis today, it can lead to infection in humans, therefore monitoring the expansion of this bacterium within the herd is important.

Recently, the research on immunoreactive proteins has been developing. These proteins are being considered as, above all, future components of ELISA (Bu et al., 2015; Bu et al., 2017), immunochromatographic assays, but also components of vaccines against mastitis caused by the most common bacterial species (Dego et al., 2021). In this article, we have described 13 proteins and most of them were involved in energy metabolism and other cellular functions.

In our study, we identified four proteins representing *S. uberis*, among which elongation factor Tu (SU 1) and glyceraldehyde-3-phosphate dehydrogenase (SU 5) are the most frequently described immunoreactive proteins (Zadoks et al., 2005; Demon et al., 2013). They are also described as cytosolic proteins; however, recent studies have indicated their multifunctional role. Therefore, they can be also present on the surface of bacterial cells and participate in pathogenesis (Amblee and Jeffery, 2015; Dego et al., 2021). Other proteins described in the context of bovine mastitis were, among others: fructose-bisphosphate aldolase, lactoferrin binding protein, glutamine synthetase, and glutamine binding protein (Collado et al., 2016; Dego et al., 2021), and the results partially correspond to our observations. tRNA-5-carboxymethylaminomethyl-2-thiouridine synthesis protein MnmG (SU 3), also described as GidA, is a bacterial and mitochondrial conserve protein, which participate in tRNA modification (Moukadiri et al., 2009). MnmG is also known as a virulence factor (Silva et al., 2021) and, as Charbonneau et al. showed, it is crucial for *in vitro Streptococcus equi* growth (Charbonneau et al., 2017). It had been shown that this protein is also important for survival in other *Streptococcus* species: *S. agalactiae* and *S. pyogenes* (le Breton et al., 2015; Hooven et al., 2016). Moreover, it had been proved that deletion of genes coding MnmG and MnmE (another enzymatic protein, which forms heterotetrameric complex with MnmG) led to reduction in biofilm formation of up to 50% in *Streptococcus mutans* (Li et al., 2014) Silva et al., by using whole-genome sequencing method for *S. uberis* characteristics, demonstrated the presence of this protein in 80% of strains obtained from cows with mastitis (Silva et al., 2021). On the other hand, GTPase Obg (SU 4) is described as a protein crucial for bacterial growth and plays an important role in bacterial stress response, however, the mechanism is unknown (Kumar et al., 2017). Nothing is known about its role in immunoreactivity in *S. uberis* isolated from cows with bovine mastitis, which may indicate the pioneer nature of our discovery.

Immunoreactive proteins of *S. agalactiae* have been widely studied with particular emphasis on humans (newborns and GBS carriage) and a type of fish belonging to the *Nile tilapia* species. In our previous paper, we also described some of them for humans (Brzychczy-Wloch et al., 2013; Dobrut et al., 2018; Pyclik et al., 2018; Dobrut and Brzychczy-Włoch, 2022), and some of them, such as elongation factor Tu (GBS 2), were also detected in this study in cows. Apart from EF-Tu, another elongation factor – elongation factor G (GBS 4), had been identified in a mixture of immunoreactive proteins isolated from bovine *S. agalactiae*. Both EF-Tu and EF-G play an important role in prokaryotic protein synthesis, and while EF-Tu is involved in translation and in the elongation phase of protein synthesis, EF-G is responsible for catalyzing movement of RNA transfer as well as mRNA in ribosomes during the translocation step by using energy stored in GTP (Chen et al., 2016). Another identified protein, 60kDa chaperonin (GBS 3) belongs to the chaperonin family, as well as GroEL, which had been identified in our previous study (Dobrut et al., 2018). Its crucial role is the involvement in folding *de novo* emerging proteins. Chaperonins are also described as virulence factors (Dobrut and Brzychczy-Włoch, 2022). Moreover, the homologs of 60 kDa chaperonin demonstrated strong immunoreactivity for *Mycobacterium tuberculosis* (Kong et al., 1993). This result corresponds with the results obtained in our previous studies, in which GroEL showed significant immunoreactivity to antibodies present in umbilical cord blood (Dobrut et al., 2018). Proteins such as surface-associated proteins Sip, phosphoglycerate kinase (Pgk) and fibronectin (FbsA) of *S. agalactiae* have been studied as components of indirect ELISA for the diagnosis of bovine mastitis (Bu et al., 2017). These proteins have also been studied in the context of vaccines providing protection from bacterial infections (Zhai et al., 2021). There is not much data on the remaining proteins, which may encourage researchers to consider them in further proteomic studies on innovative biomarkers or components of subunit vaccine offering protection from bovine mastitis caused by *S. agalactiae*.

For *E. coli*, the immunoreactive protein most frequently described is the outer membrane protein A (ompA), which has been identified as protein no. EC 3 in our studies. OmpA is a major constituent of the outer membrane of this species and has been studied in the context of its immunoreactivity and the results correspond with the ones obtained in our investigation (Todhunter et al., 1991; Rainard et al., 2017; Chen et al., 2020). For its immunogenic role, another protein belonging to the omp family, the outer membrane protein F, has been described (Wang et al., 2017). Also, fimbrial adhesin factor, which belongs to the F17 family, has been described as a promising immunodominant antigen (Chen et al., 2017). The other two identified immunoreactive proteins, molybdenum cofactor biosynthesis protein B (EC 1) and aldehyde reductase YahK (EC 2), the former is responsible for the biosynthesis of molybdopterin, demonstrates an ability to bind GTP, has low GTPase activity (Eaves et al., 1997), catalyzes the reduction of reductases into an appropriate alcohol and is a major source of NADPH-dependent aldehyde reductase activity in *E. coli* (Pick et al., 2013). To our best knowledge, they have not been described in the context of their immunoreactivity to *E. coli* in bovine mastitis. That may also point to the innovative nature of our investigation.

In our paper, we aimed to detect surface proteins, which could be easily accessed to detect antibodies in the lateral flow assay, and thus perform the examination rapidly, directly in the cowshed. Undoubtedly, according to *in vitro* results followed by bioinformatic predictions, with particular emphasis on its predicted localization among the bacterial cell, the most promising protein out of all the examined species is ompA (EC 3) isolated from *E. coli*, however, the remaining proteins can also be included in further investigation, as both *in vitro* and *in silico* analyses allowed us to assume that all of the proteins described can be associated with bacterial protein surface. A culture of the pellet remaining after the protein isolation procedure showed bacterial growth in every examined isolate – even though determined qualitatively. Thus, it can be hypothesized that the protein isolation procedure based on SDS had not disrupted the bacterial cell wall, and only the cell surface-associated protein had been isolated. We are aware that culturing the bacterial cell pellet may not directly confirm that proteins subjected to further investigation were the only cell surface-associated ones. There is a possibility that, even though the protein isolation procedure according to which we obtained proteins for the experiments was dedicated to surface proteins, the bacterial membranes could have been disrupted. However, according to numerous publications, the identified proteins are described as multifunctional, and hence their localization among the bacterial cell can vary and they can be present both inside and outside the cell. Nevertheless, with no doubts, it requires further investigation such as experiments carried out with whole bacterial cells and monoclonal antibodies specific to the examined proteins.

While it is worth highlighting, the proteins mentioned above did not react or barely reacted with negative samples. It can be hypothesized that any reactivity may be connected with the fact that the animals, from which the blood samples were collected, had previously come into contact with the studied bacterial species and therefore, even though these bacteria were not present in milk samples and did not present any clinical signs of infection, some reactivity was noticed. To our best knowledge, most of the bacterial proteins identified and described in this article have not been described as immunoreactive with bovine isolates; thus, according to their confirmed immunoreactivity, both *in vitro* and *in silico*, and regarding their surface-associated localization, they can be considered as potential biomarkers in immunodiagnostic assays. We are aware of the limitations of the study in the form of the number of bacterial strains, which among others, may lead to difficulties in determination of protein conservativity for particular proteins; however, the results stem from the framework of the project according to which the research was carried out. We studied one to three samples, which originated from various animals, from different herds, and even from different geographical regions. We cannot disagree that the number of probes is not enough; however, the aim of the study was to present the potential candidates, which should be studied in further studies, on an extended number of samples, in the presence of defined, monoclonal antibodies, to confirm their statistically significant specificity and sensitivity in immunodiagnostic assay. However, we believe that, due to the lack of similar sufficient data, our results will deepen the knowledge in this area and encourage further investigation.

## Data availability statement

The data presented in the study are deposited in the MassIVE repository, accession number MSV000091181.

## Ethics statement

Ethical review and approval was not required for the animal study because In the opinion of 1 of the Local Ethics Committee for Animal Experiments, approval of the studies described in the manuscript is not required.

## Author contributions

AD designed the concept of the study, obtained funds for research, coordinated the project, analyzed results, drafted the manuscript, DW-G carried out bacterial protein isolation, conducted detection of the immunoreactive proteins, AM carried out bioinformatic analyses, DP-O and KM carried out protein identification, AT collected milk and serum samples, UM collected milk samples, MB-W was a supervisor and helped to draft the manuscript. All authors read and approved the final manuscript.
